# Identification and verification of diagnostic biomarkers in recurrent pregnancy loss via machine learning algorithm and WGCNA

**DOI:** 10.3389/fimmu.2023.1241816

**Published:** 2023-08-25

**Authors:** Changqiang Wei, Yiyun Wei, Jinlian Cheng, Xuemei Tan, Zhuolin Zhou, Shanshan Lin, Lihong Pang

**Affiliations:** ^1^ Department of Prenatal Diagnosis, The First Afliated Hospital of Guangxi Medical University, Nanning, Guangxi, China; ^2^ Guangxi Key Laboratory of Thalassemia Research, Nanning, Guangxi, China; ^3^ National Health Commission Key Laboratory of Thalassemia Medicine (Guangxi Medical University), Nanning, Guangxi, China; ^4^ Key Laboratory of Early Prevention and Treatment for Regional High Frequency Tumor (Guangxi Medical University), Ministry of Education, Nanning, Guangxi, China; ^5^ Guangxi Key Laboratory of Early Prevention and Treatment for Regional High Frequency Tumor, Nanning, Guangxi, China

**Keywords:** recurrent pregnancy loss, diagnostic biomarkers, machine learning, WGCNA, immune cell infiltration

## Abstract

**Background:**

Recurrent pregnancy loss defined as the occurrence of two or more pregnancy losses before 20-24 weeks of gestation, is a prevalent and significant pathological condition that impacts human reproductive health. However, the underlying mechanism of RPL remains unclear. This study aimed to investigate the biomarkers and molecular mechanisms associated with RPL and explore novel treatment strategies for clinical applications.

**Methods:**

The GEO database was utilized to retrieve the RPL gene expression profile GSE165004. This profile underwent differential expression analysis, WGCNA, functional enrichment, and subsequent analysis of RPL gene expression using LASSO regression, SVM-RFE, and RandomForest algorithms for hub gene screening. ANN model were constructed to assess the performance of hub genes in the dataset. The expression of hub genes in both the RPL and control group samples was validated using RT-qPCR. The immune cell infiltration level of RPL was assessed using CIBERSORT. Additionally, pan-cancer analysis was conducted using Sangerbox, and small-molecule drug screening was performed using CMap.

**Results:**

A total of 352 DEGs were identified, including 198 up-regulated genes and 154 down-regulated genes. Enrichment analysis indicated that the DEGs were primarily associated with Fc gamma R-mediated phagocytosis, the Fc epsilon RI signaling pathway, and various metabolism-related pathways. The turquoise module, which showed the highest relevance to clinical symptoms based on WGCNA results, contained 104 DEGs. Three hub genes, WBP11, ACTR2, and NCSTN, were identified using machine learning algorithms. ROC curves demonstrated a strong diagnostic value when the three hub genes were combined. RT-qPCR confirmed the low expression of WBP11 and ACTR2 in RPL, whereas NCSTN exhibited high expression. The immune cell infiltration analysis results indicated an imbalance of macrophages in RPL. Meanwhile, these three hub genes exhibited aberrant expression in multiple malignancies and were associated with a poor prognosis. Furthermore, we identified several small-molecule drugs.

**Conclusion:**

This study identifies and validates hub genes in RPL, which may lead to significant advancements in understanding the molecular mechanisms and treatment strategies for this condition.

## Introduction

Recurrent pregnancy loss (RPL) is defined as two or more clinically confirmed pregnancy failures occurring before 20-24 weeks of gestation. It is a significant complication affecting around 2.5% of women during pregnancy (1). The exact etiology of RPL remains unknown, although it is primarily believed to be associated with uterine anatomical, genetic, and immunological abnormalities (2). Recent studies have identified additional factors potentially linked to RPL, including endometritis and genetic susceptibility to embolism. Targeted treatments for specific etiologies, including early pregnancy administration of aspirin, heparin, and other medications, as well as surgical management of uterine anomalies, have shown improved pregnancy outcomes for some RPL patients (3). Nevertheless, approximately 50% of RPL cases have an unknown etiology (4), and although clinical empirical treatment with progesterone supplementation may benefit certain patients (5), further randomized controlled trials are necessary to validate these findings. Therefore, investigating the diagnostic genes and pathogenesis of recurrent pregnancy loss holds significant value.

With the rapid development of transcriptomics, bioinformatics analysis based on gene expression profile data to explore signature biomarkers has been widely performed (6, 7). The Gene Expression Omnibus (GEO) database (https://www.ncbi.nlm.nih.gov/gds) of the National Center for Biotechnology Information (NCBI) provides comprehensive expression profiles for a wide range of diseases, enabling researchers to conduct diverse data analyses. Through their analysis of the GEO dataset, Chen Y et al. identified Atp6v1g3 as a key gene associated with recurrent spontaneous abortion (8). In another study, researchers conducted data analysis along with *in vivo* and *in vitro* experiments and discovered that exosomal miR-205 can induce angiogenesis in ovarian cancer through the PTEN-AKT pathway, revealing a potential therapeutic target for the disease (9). Seven genes closely associated with recurrent miscarriage were screened and experimentally validated by Wei Peiru et al. using single-cell sequencing technology combined with comprehensive bioinformatics analysis, which may have potential value in predicting this disease (10).

Weighted co-expression network analysis (WGCNA), developed in 2008 by Peter Langfelder and Steve Horvath (11), allows the identification of co-expressed gene modules and the exploration of associations between gene networks and clinical phenotypes based on gene expression. It is widely employed for the discovery of disease biomarkers (12, 13). Machine learning, a special class of algorithms that enable computers to learn from data and make predictions autonomously, represents an emerging field in medicine with the potential to significantly contribute to the exploration of disease mechanisms and clinical diagnosis (14, 15).

Immune cell imbalance plays a significant role in the pathogenesis of numerous diseases (16–18). The maternal-fetal interface consists of various immune cells, including metaphase natural killer cells, macrophages, T cells, dendritic cells, B cells, and NKT cells (19). Immune imbalance is one of the etiologies leading to recurrent spontaneous abortion. Therefore, conducting the immune cell infiltration analysis can provide valuable insights into the pathogenesis of RPL.

In this study, we utilized WGCNA and three machine learning algorithms: LASSO, SVM-RFE, and Random Forest, to screen for diagnostic biomarkers of RPL. Subsequently, scoring models were developed using artificial neural networks to assess their diagnosis values. Through PCR experiments, we verified the abnormal mRNA expression levels of hub genes in RPL. Moreover, CIBERSORT was employed to determine the infiltration of immune cells in the RPL and control groups, and the correlation between hub genes and immune cells was explored. Additionally, pan-cancer analysis of the hub gene was conducted based on the TCGA database, aiming to explore the role of hub genes in RPL and various cancers. These works are expected to provide novel insights into the study of RPL.

## Materials and methods

### Datasets and patient selection

The raw data of GSE165004 was downloaded from the GEO database (20), based on the GPL16699 platform, including endometrial samples from 24 patients with RPL, 24 patients with unexplained infertility, and 24 healthy fertile women (controls). For this study, we only included 24 RPL patients and 24 healthy fertile women. Additionally, we downloaded GSE183555 (21), based on the GPL21697 platform, which consisted of 5 cases each of RPL patients and healthy fertile women, used as a validation dataset. The detailed information of the dataset is presented in **Table 1**.

**Table 1:** Baseline characteristics of GEO datasets used in this study.

GSE series	Platforms	Type	Sample size	Timing of biopsy
GSE165004	GPL16699	mRNA	48 (24 RPL samples and 24 control samples)	days 19-21 of the menstrual cycle
GSE183555	GPL21697	mRNA	10 (5 RPL samples and 5 control samples)	days 21-23 of the menstrual cycle

Decidua samples were collected from 20 women who underwent selective termination of pregnancy and 20 women with RPL at the Department of Obstetrics and Gynecology, the First Affiliated Hospital of Guangxi Medical University, to verify the expression of the hub gene in RPL. The study was conducted in accordance with the principles outlined in the Declaration of Helsinki and received ethical approval from the Research Ethics Committee of the First Affiliated Hospital of Guangxi Medical University (No. 2023-K057-01). The inclusion criteria for patients were as follows: RPL group: 1. a history of two or more unexplained pregnancy losses. 2. Absence of fetal heartbeat detected by ultrasound between 6-8 weeks of gestation. Control group: 1. Spontaneously conceived pregnancies with a duration of 6-8 weeks and voluntary termination for non-medical reasons; 2.No history of spontaneous abortion or clinical signs of threatened abortion. 3. Fetal cardiac activity is observed on ultrasound within 3 days before pregnancy termination. Exclusion criteria: 1. Abnormal embryonic chromosomal karyotype analysis; 2. Known causes of miscarriage; 3. and patients with concurrent medical conditions.

After induced abortion, decidua tissues were transferred to a curved dish using forceps within 15 minutes. The tissues were thoroughly rinsed with pre-cooled physiological saline to remove surrounding blood clots. Approximately 3g of the sample was separated using a surgical knife and placed in Trizol (Takara, Japan) for RT-PCR experiments. Another portion of approximately 10g was placed in a cryotube and stored in a liquid nitrogen tank for subsequent experiments.

Patients who were enrolled in the study signed the relevant informed consent before surgery, and the general baseline data of patients in both groups are presented in **Table 2**.

**Table 2:** General baseline data of patients.

	Control(n=20)	RPL group(n=20)	p
Age(years)	33.85 ± 4.320	34.50 ± 4.65	0.65
Gestational Weeks(weeks)	7.72 ± 0.99	7.54 ± 0.85	0.53
BMI(kg/m^2^)	21.76 ± 3.51	21.66 ± 2.79	0.92

*BMI, body mass index.

### Data preprocessing and differentially expressed genes (DEGs) identificating

GSE165004, based on the Agilent-039494 SurePrint G3 Human GE v2 8x60K microarray, was RMA background corrected, normalized, and log2 transformed using the affy package (22) in R. The results before and after standardization are presented in box plots (**Supplementary Figure 1**). GSE183555 was downloaded from the GEO database as a validation dataset.

Probe annotation was performed on standardized expression profiles, excluding probes without matching gene symbols. For genes with multiple probes, the average value was calculated as their expression values. Differential expression analysis was performed using the limma package (23) with screening criteria of |log2 FC| > 0.585 and p-value < 0.05. Heat maps and volcano plots were visualized using the “heatmap” and “ggplot2” packages.

### Functional enrichment analysis

Gene Ontology (GO) term and Kyoto Encyclopedia of Genes and Genomes (KEGG) pathway enrichment analysis of DEGs were performed using the “clusterProfiler” package (24). Meanwhile, c2.cp.kegg.v7.4.symbols.gmt was selected as the reference gene set, and Gene Set Enrichment Analysis (GSEA) was also conducted. A p-value of less than 0.05 was set as the criterion for significant enrichment.

### Co-expression network analysis and module selection

The “WGCNA” package was used to construct a co-expression network, which can associate gene networks with clinical features. First, a scale-free co-expression network was constructed, followed by the transformation of the matrix into an adjacency matrix and a TOM matrix. Then, the dissimilarity of the TOM matrix (dissTOM) was calculated and at least 60 genes were clustered into different modules using a dynamic tree cutting algorithm. Eigengene was calculated and the abline was set to 0.25 to merge similar modules on the clustering tree. Finally, clinical information was integrated with the modules to identify the most relevant modules to RPL through Pearson correlation analysis.

### Hub genes screening via machine learning

Three machine learning algorithms, LASSO, SVM-RFE, and Random Forest, were used to analyze the DEGs of the most critical modules in the WGCNA results to screen significant variables. LASSO regression considers both the goodness of fit of regression coefficients and the absolute magnitude of those coefficients for effective feature selection. The “glmnet” package was used for performing LASSO regression analysis (25). SVM-RFE is a posterior term selection algorithm for sequences based on the maximum interval principle of SVM, which is one of the most widely used and superior feature selection algorithms. In this study, SVM-RFE analysis was performed using “e1071”, “kernlab”, and “caret” packages (26). The RandomForest algorithm integrates numerous decision trees to form a forest, is employed for predicting the final outcome. In this study, the RandomForest analysis was conducted using “randomForest” packages. The outputs of the three algorithms were intersected utilizing a Venn diagram (27), and the common results were identified as hub genes.

### The artificial neural network prognostic model

Artificial neural networks (ANNs) can accurately construct diagnostic models by simulating human brain information processing patterns to correctly classify complex data (28). Using the “neuralnet” and “NeuralNetTools” packages, the stable ANN model was constructed by scoring the hub genes. The discriminative ability of hub genes between experimental and control groups, as well as the accuracy of ANN model in the test and validation sets, were evaluated using receiver operating characteristic (ROC) curves.

### Quantitative real time polymerase chain reaction (RT-qPCR)

Total RNA was extracted from decidua tissues using Trizol (Takara, Japan). RNA purity and concentration were determined using NanoDrop 2000 (Thermo Fisher Scientific, USA). Genomic DNA was removed using the Prime Script RT reagent kit (Takara, Japan). Subsequently, RNA was reverse transcribed into cDNA. The PCR reaction was performed using the SYBR Green Master Mix kit (Qiagen, Germany) with cDNA as the template and human β-actin (H-ACTB) as the internal reference. The primers were designed and synthesized by Sangon Biotech Co., Ltd(China), and the primer sequences are shown in **Table 3**. Before conducting experiments, primer specificity was assessed using NCBI’s Primer-BLAST to exclude potential non-specific matches. Meanwhile, during qPCR, primer specificity was ensured by observing the melting curves of the reactions. The relative expression of hub gene mRNA was calculated using the 2-ΔΔCT method. The experiment was conducted with at least three biological and three technical replicates. In each replicate well of the sample, a difference in CT values within 0.5 is considered eligible for analysis.

**Table 3:** The primers of hub genes and β-Actin.

Gene name	Primer orientation	Sequences
ACTR2	Forward	GGCAGTTCTGACTTTGTACGC
ACTR2	Reverse	CCAGTCTCCTGGTAAGATGAGG
WBP11	Forward	CCAGTGCAACAGCCACAATTA
WBP11	Reverse	ACGTTCAAAGGTTTCACGCAG
NCSTN	Forward	AATGTGAGCTATCCCGAATG
NCSTN	Reverse	GCGATGTAATGTTGAAGAGGC
β-Actin	Forward	TGCCACCCAGCACAATGAA
β-Actin	Reverse	CTAAGTCATAGTCCGCCTAGAAGCA

### Immune cell infiltration analysis

Since its first publication in Nature Methods in 2015, CIBERSORT has been utilized for estimating the abundance of immune cell types using gene expression data (29). Using the CIBERSORT package and LM22, an official background file of cellular gene sets, we calculated the proportions of 22 immune cell types in RPL and displayed them in a bar chart. Differences in immune cells between RPL and controls were visualized using the “vioplot” package. The “corrplot” package was used to create a heatmap depicting the correlation of immune cells. Additionally, we analyzed the correlation of hub genes with immune cells.

### Pan-cancer analysis

The normalized pan-cancer datasets, including TCGA,TARGET and GTEx, were obtained from the UCSC database (https://xenabrowser.net/) via the Sangerbox website (30). The expression data of the hub genes in each sample were extracted, and the expression values were log2(x+0.001) transformed. Differential analysis was conducted using unpaired Wilcoxon tests. The Cox proportional hazards regression model was constructed using the survival package to analyze the relationship between gene expression and prognosis in each tumor (31). The Logrank test was used for the comparison of prognostic significance. Additionally, we assessed the association between hub genes and overall survival (OS) in pan-cancer using Kaplan-Meier plots.

### Potential drug prediction

The Connectivity Map(CMap) (https://clue.io) is a database for studying the correlation between gene expression and small molecule drugs, which can help researchers to screen for highly relevant drugs for diseases. We uploaded the list of DEGs of RPL to the CMap database and selected the “Latest” version for analysis. In the results, compounds with negative scores may potentially contribute to the treatment of the disease, and we screened compounds with norm_cs < -1.50 as potential drugs for RPL treatment.

### Statistical analysis

Statistical analysis of this study was based on R software (version 4.3.0). The “ggplot2” and “ggpubr” packages were utilized for data visualization. The normality of the data was assessed using the Shapiro-Wilk test, and for normally distributed continuous variables, such as patient age, gestational weeks, and body mass index (BMI), analysis was performed using the Student’s t-test. The Wilcoxon test was employed to compare the mRNA expression levels(Non-normally distributed variables) of individual genes between the RPL and control groups. The “pROC” was employed to evaluate the efficacy of the hub genes and ANN model. A p-value less than 0.05 was considered indicative of statistical significance in all statistical analyses.

## Results

### Identification of DEGs in RPL and functional enrichment analysis

**Figure 1:**
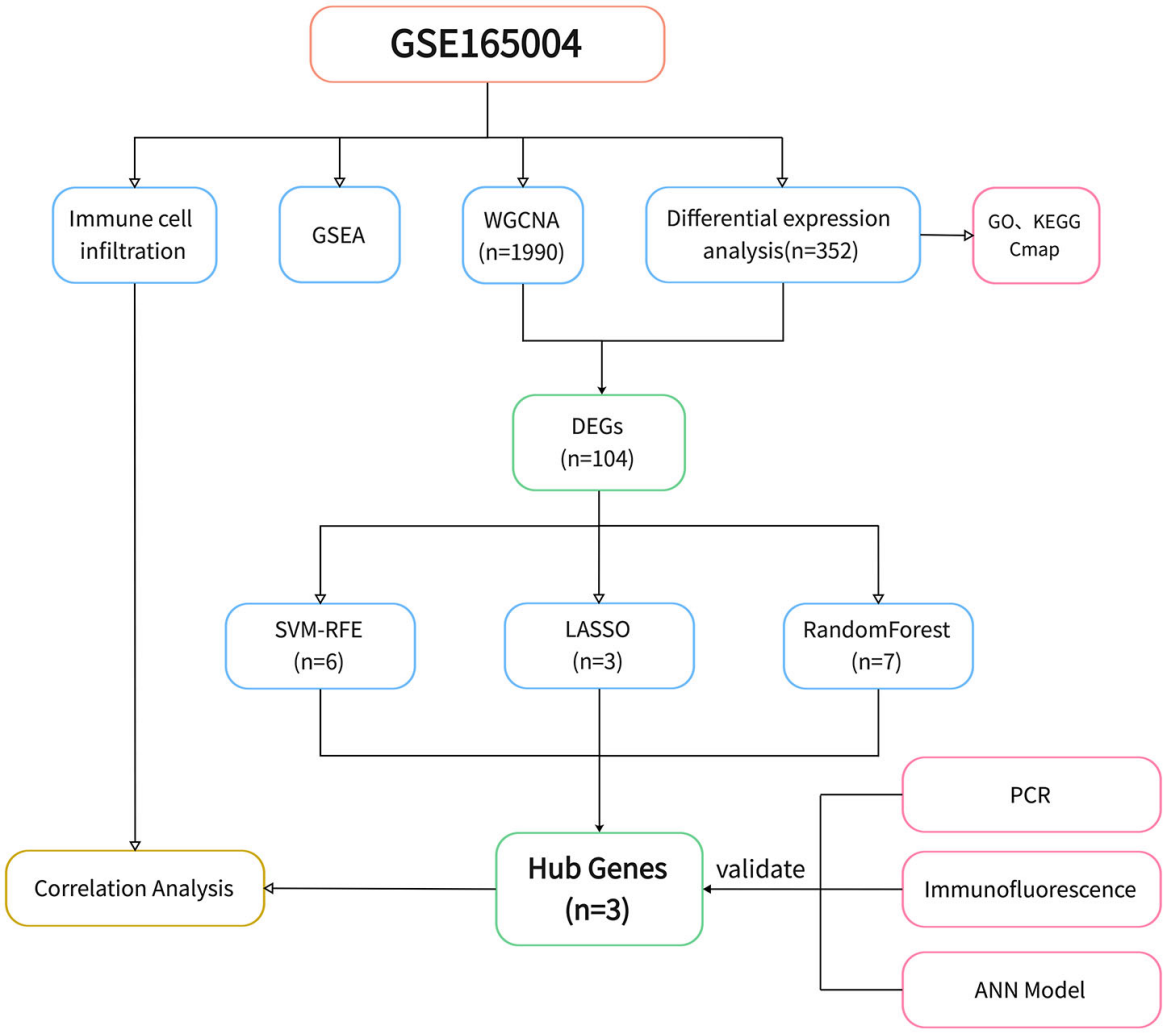
Flowchart of study design.

**Figure 2:**
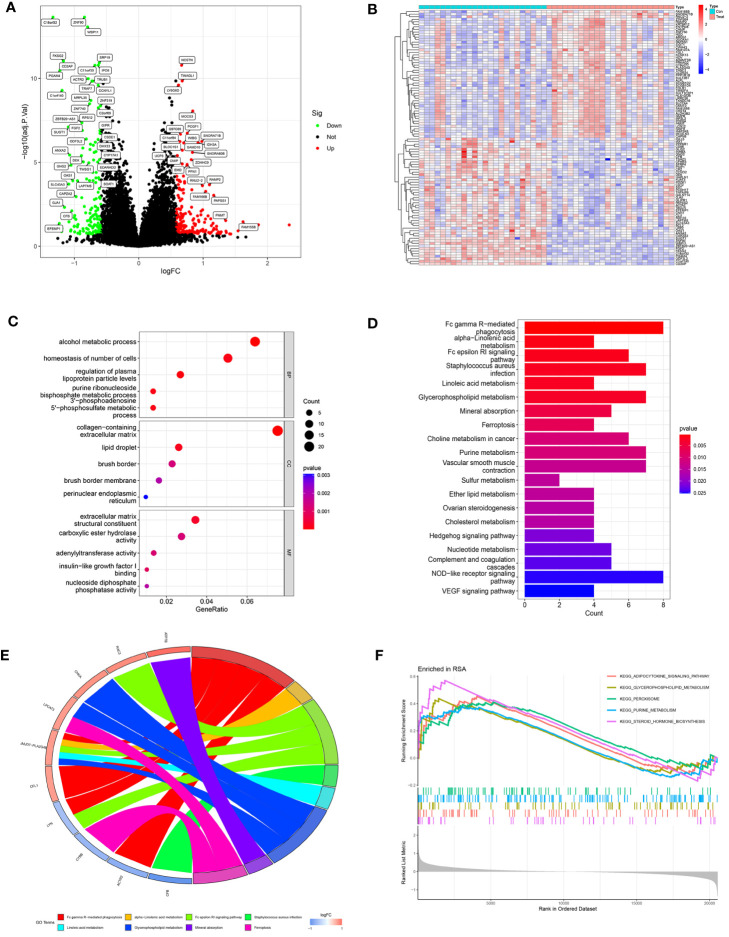
Analysis of DEGs in RPL patients and healthy controls. **(A)** Volcano plot of DEGs. The red dots represent the up-regulated genes and the green dots represent the downregulated genes, while gray dots represent nonsignificant genes. **(B)** Heat map of the top 100 differentially expressed genes. **(C)** GO enrichment analysis. (BP, biological process; CC, cellular component; MF, molecular function). **(D)** KEGG pathway enrichment analysis. **(E)** Main KEGG pathways and related genes. **(F)** GSEA results in the RPL group.

The study design is shown in **Figure 1**. After data processing of GSE165004 (**Supplementary Figures 1A, B**), differential expression analysis was performed and a total of 198 up-regulated genes and 154 down-regulated genes were obtained. The results were presented in volcano plot (**Figure 2A**) and heatmaps (**Figure 2B**). To understand the functions and potential pathways involved in these DEGs, functional enrichment analysis was conducted. The results of GO analysis showed that in biological processes, these genes are mainly involved in alcohol metabolic process, homeostasis of cell number, and regulation of plasma. In terms of cell component, these genes are mainly enriched in collagen-containing extracellular matrix, lipid droplet, and brush border. As for molecular function, these genes were mainly enriched in extracellular matrix structural constituent, carboxylic ester hydrolase activity, and adenylyltransferase activity (**Figure 2C**).

KEGG results revealed that DEGs are mainly involved in Fc gamma R-mediated phagocytosis, Fc epsilon RI signaling pathway, and various metabolic pathways (alpha-Linolenic acid metabolism, Linoleic acid metabolism, Glycerophospholipid metabolism, Purine metabolism) (**Figure 2D**).

The chord diagram illustrated the intricate mapping relationships between genes and pathways. Notably, the KEGG pathway associated with JMJD7-PLA2G4B showed the highest abundance (**Figure 2E**), suggesting it may play a key role in bridging different pathways. GSEA results exhibited that the pathways significantly enriched in RPL were Purine metabolism, Glycerophospholipid metabolism, Adipocytokine signaling pathway, Peroxisome, and Pteroid hormone biosynthesis (**Figure 2F**). These findings indicate that metabolic pathway disorders may have a close relationship with RPL pathogenesis.

### WGCNA results

**Figure 3:**
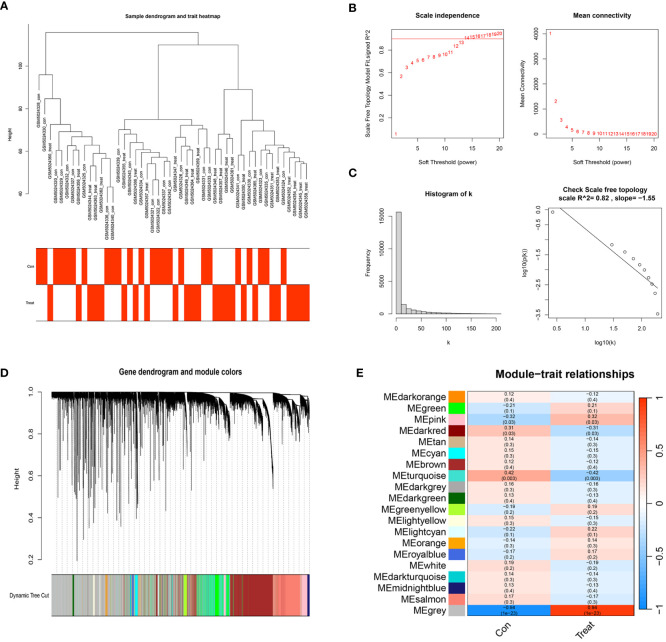
WGCNA co–expression network. **(A)** Sample clustering dendrogram of 48 samples of GSE165004. **(B)** The scale-free fit index for various soft-thresholding powers (β) and the mean connectivity for various soft-thresholding powers. **(C)** Histogram of connectivity distribution and the scale-free topology when β=13. **(D)** Dendrogram of genes clustered via the dissimilarity measure (1-TOM). **(E)** Heatmap of the correlation between genes and clinical traits.

We selected a total of 20,552 genes from GSE165004 for WGCNA and drew a sample dendrogram and trait heat map (**Figure 3A**). β=13 (R2 = 0.82) was selected as a suitable soft threshold for the construction of the scale-free network **(** **Figures 3B, C**). Modules were hierarchically clustered based on the TOM matrix and similar modules on the clustering tree were merged (**Figure 3D**). Twenty co-expression modules were obtained and **Figure 3E** showed the correlation of these modules with clinical information. The results showed that the turquoise module was most closely related to RPL (r=-0.42, p=0.003) and contained a total of 1,990 genes.

### Identification of hub biomarkers

**Figure 4:**
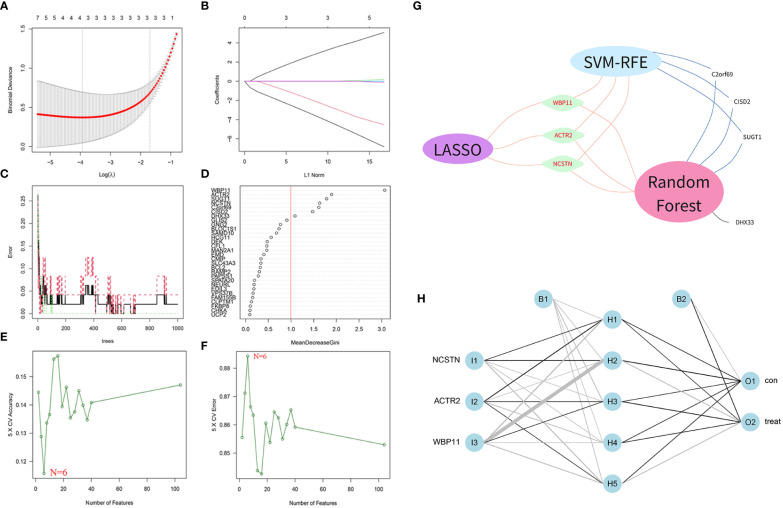
Screening diagnostic biomarkers based on machine learning. **(A, B)** The variables selection in LASSO model(n=3). **(C, D)** Optimal biomarkers screening by SVF-RFE algorithm(n=6). **(E, F)** Significant feature selected via the random forest algorithm(n=7). **(G)** Venn diagram of overlapping genes in three algorithm. **(H)** Schematic presentation of the ANN model.

A total of 104 DEGs were included in the turquoise module, with 64 up-regulated and 40 down-regulated. Machine learning algorithms were employed to identify hub genes from the pool of 104 DEGs. In the LASSO regression analysis, the optimal lambda value of 0.0197 was determined after ten cross-validations and three hub genes(WBP11, ACTR2 and NCSTN) were extracted (**Figures 4A, B**). In the SVM-RFE, the classifier error was minimized when the number of features was 6, and WBP11, SUGT1, CISD2, ACTR2, C2orf69 and NCSTN were screened as hub features (**Figures 4C, D**). Random forest results identified seven genes with importance scores >1.0 as potential diagnostic biomarkers for RPL: WBP11, ACTR2, SUGT1, NCSTN, C2orf69 CISD2, and DHX33 (**Figures 4E, F**). In total, three biomarkers overlapped between these three algorithms (ACTR2, NCSTN, and WBP11) (**Figure 4G**). Artificial neural network models were constructed basing on the three hub genes (**Figure 4H**).

### Diagnostic efficacy of hub biomarkers and ANN model

**Figure 5:**
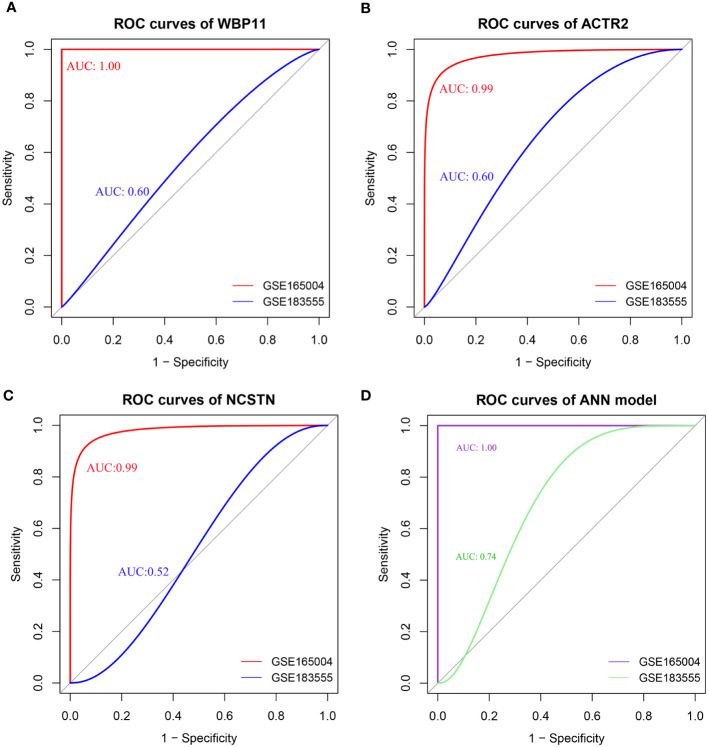
**(A–D)** ROC of individual hub gene(WBP11, ACTR2, and NCSTN) and ANN model, the curve is optimized using the smooth() function.

The diagnostic efficacy of the 3 hub genes was evaluated using ROC curves. As shown in **Figure 5**, the area under the curve (AUC) values of the ROC curves for WBP11 were 1.00 and 0.60 in the test set GSE165004 and validation set GSE183555 (**Figure 5A**). The AUC values for ACTR2 were 0.99 and 0.60 (**Figure 5B**), and for NCSTN were 0.99 and 0.52 (**Figure 5C**).

The ROC results showed that the ANN model could effectively discriminate between RPL and controls, with an AUC value of 1.00 for the ROC curves for the test set GSE165004 and an AUC value of 0.74 for the validation set GSE183555 (**Figure 5D**). These findings suggest that the combined use of the three biomarkers contributed to higher diagnostic accuracy.

### Verification of hub biomarkers

**Figure 6:**
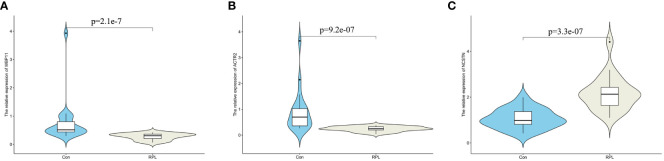
The mRNA expression validation between normal and RPL tissues by RT-qPCR. **(A)** WBP11; **(B)** ACTR2; **(C)** NCSTN.

To verify the mRNA and protein expression of the hub gene, decidua samples were collected from 20 control patients and 20 RPL patients. RT-qPCR results showed that the relative expression of WBP11 mRNA in the RPL group was lower than that in the control group (**Figure 6A**). The relative expression of ACTR2 mRNA was also lower than that of the control group, while the relative expression of NCSTN mRNA was higher than that of the control group (**Figures 6B, C**).

### Immune cell infiltration results

**Figure 7:**
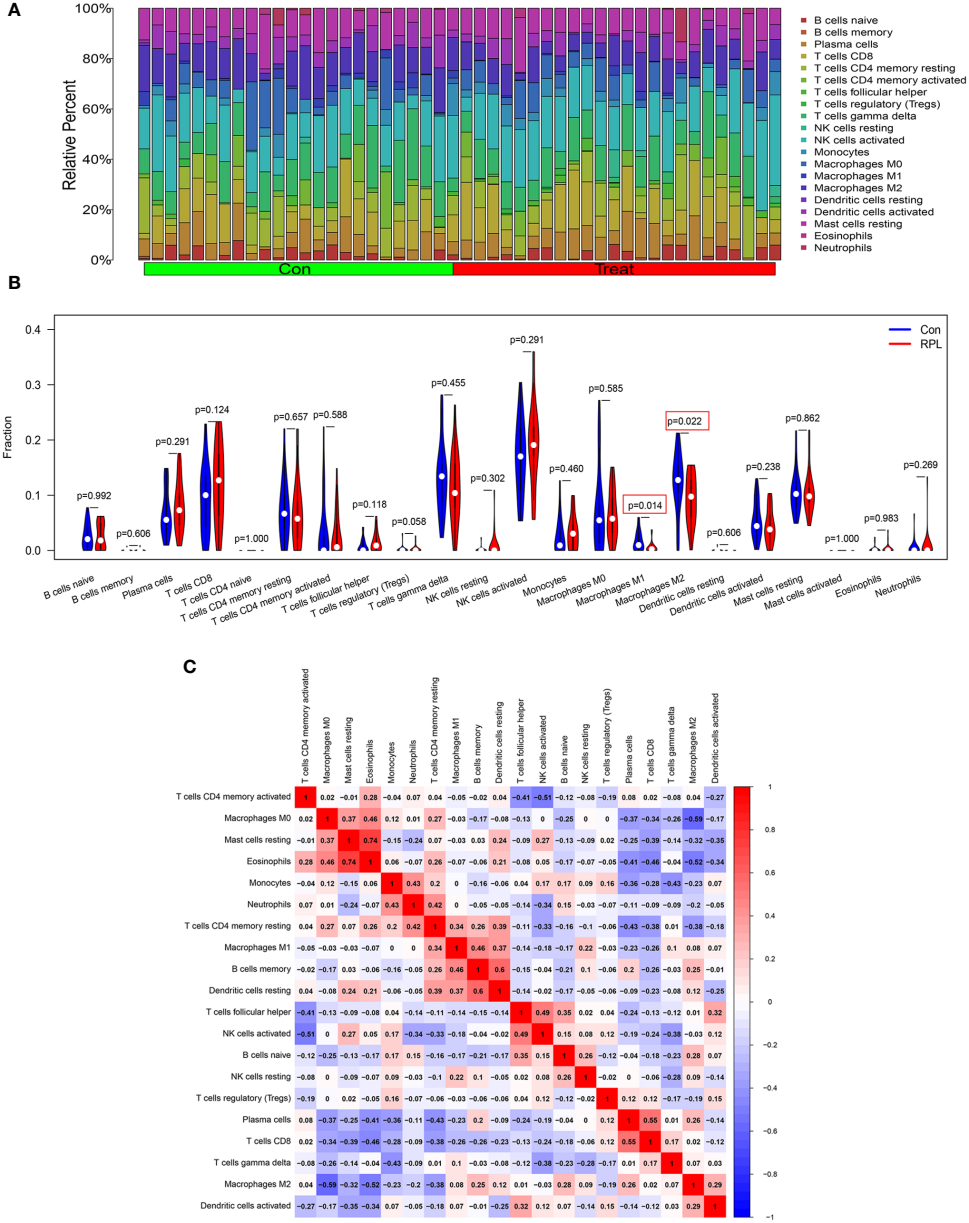
Landscape of 22 different immune cell infiltration in RPL. **(A)** Relative proportions of immune cell infiltration. **(B)** The diagram of the difference in immune cell infiltration proportion between the RPL and control groups. **(C)** The correlation heatmap among immune cell populations, blue and red indicate positive and negative correlation, respectively.

We further investigated immune cell infiltration in RPL and control groups using CIBERSORT (**Figure 7A**). The infiltration levels of M1 and M2 macrophages were significantly lower in the RPL group compared to the control group (**Figure 7B**). Notably, the infiltration of both CD4 naïve T cells and activated mast cells were not detected in the results. Correlation analysis revealed a strong positive correlation between Eosinophils and resting mast cells (r=0.74), while M0 macrophages exhibited a negative correlation with M2 macrophages (r=-0.59) (**Figure 7C**).

**Figure 8:**
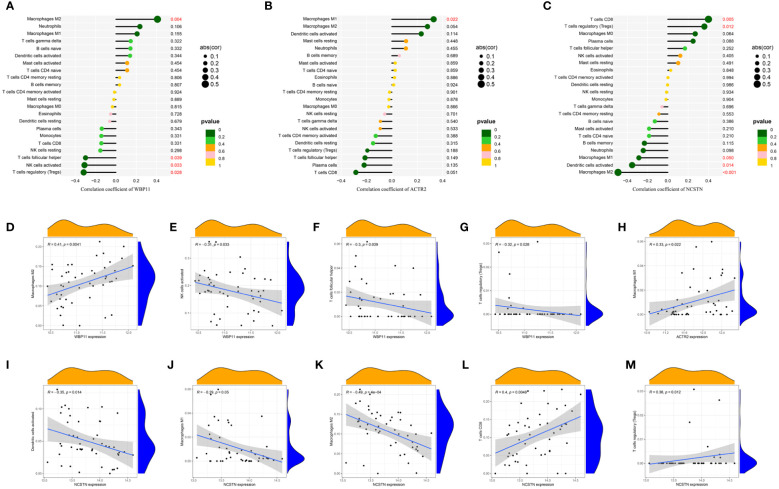
The lollipop chart showed the relationships between RPL-related hub genes and immune cell infiltration. **(A, D–G)** WBP11; **(B, H)** ACTR2; **(C, I–M)** NCSTN.

The relationship between the three hub genes and immune cells was further investigated using Spearman correlation analysis. The results suggested that WBP11 had a positive correlation with M2 Macrophages, while it showed a negative correlation with follicular helper T cells, activated NK cells and regulatory T cells (Tregs) (**Figures 8A, D–G**). ACTR2 was positively correlated with M1 Macrophages (**Figures 8B, H**). NCSTN exhibited a positive correlation with CD8 T cells and Tregs, and a negative correlation with activated Dendritic cells and M2 Macrophages (**Figures 8C, I–M**).

### Pan-cancer analysis results of hub biomarker

**Figure 9:**
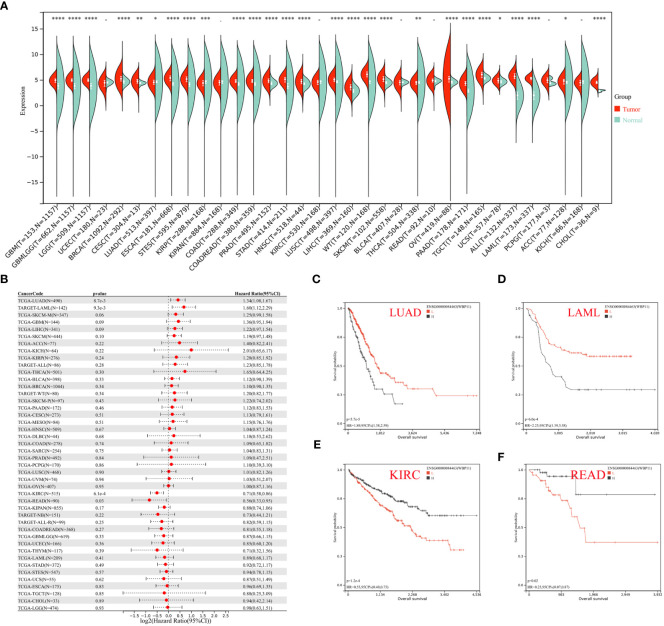
**(A)** Pan–cancer expression levels of WBP11 in the TCGA and GTEx datasets. **(B)** The correlation between WBP11 expression and OS in various tumors using Cox regression model analysis. **(C–F)** The K-M plot of WBP11 expression and several tumors, LUAD, LAML, KIRC, and READ, respectively. -: p>0.05; *: p<0.05; **: p<0.01; ***: p<0.001; ****: p<0.0001.

Expression data of three hub genes were extracted from various samples and analyzed. WBP11 was found to be significantly upregulated in 24 tumor types (GBM, GBMLGG, LGG, BRCA, CESC, ESCA, STES, COAD, COADREAD, PRAD, STAD, HNSC, LUSC, LIHC, WT, SKCM, OV, PAAD, TGCT, UCS, ALL, LAML, ACC, and CHOL), and significantly downregulated in LUAD, KIRP, and THCA (**Figure 9A**). The OS analysis (**Figure 9B**), using Cox regression, showed high expression of WBP11 in LAML and LUAD was associated with poor prognosis, while low expression of it in KIRC and READ was associated with poor prognosis (**Figures 9C–F**).

ACTR2 exhibited high expression in 28 tumor types (GBM, GBMLGG, LGG, UCEC, BRCA, CESC, LUAD, ESCA, STES, KIPAN, COAD, COADREAD, PRAD, STAD, HNSC, KIRC, LUSC, LIHC, WT, SKCM, BLCA, THCA, OV, PAAD, TGCT, UCS, LAML, CHOL), while low expression was observed in KIRP and KICH (**Supplementary Figure 2A**). In GBMLGG, LGG, LAML, CESC, LUAD, KIRP, KIPAN, LIHC, MESO, PAAD, LAML, and ACC, ACTR2 acted as a risk factor, while it acted as a protective factor in KIRC and NB (**Supplementary Figures 2B–F**).

NCSTN gene expression was upregulated in 30 tumor types, including GBM, GBMLGG, LGG, UCEC, BRCA, CESC, LUAD, ESCA, STES, COAD, COADREAD, PRAD, STAD, HNSC, KIRC, LUSC, LIHC, WT, SKCM, BLCA, THCA, OV, PAAD, TGCT, UCS, ALL, LAML, PCPG, ACC, and CHOL, while it was significantly downregulated in KICH (**Supplementary Figure 3A**). In the prognosis analysis, high expression of NCSTN was associated with poor overall survival in 12 cancers, including GBMLGG, LGG, LAML, CESC, LUAD, KIRP, KIPAN, GBM, LIHC, UVM, LAML, and PCPG, while it showed opposite results in OV (**Supplementary Figures 3B–F**).

### Small-molecule drugs related to RPL

Using CMap, we investigated small molecule drugs associated with RPL and identified a total of 17 compounds. **Table 4** displays the top 10 drugs with the highest correlation to RPL. Notably, guanaben-acetate and adoprazine exhibited a strong negative correlation with RPL, indicating their potential value in treating this condition.

**Table 4:** The top10 potential drugs of RPL screening by the CMAP database.

pert_id	pert_iname	moa	norm_cs
K62736196	guanaben-acetate	Adrenergic receptor agonist	-1.6975
K34870043	adoprazine	Dopamine receptor	-1.6239
K42728290	NVP-BGJ398	FGFR inhibitor	-1.5986
K47761761	PD-168077	Dopamine receptor agonist	-1.5944
A59808129	guggulsterone	Estrogen receptor agonist	-1.5756
K62996583	lidoflazine	Calcium channel blocker	-1.5443
K52751261	TAK-715	P38 MAPK inhibitor	-1.5334
A75455249	kavain	Calcium channel blocker	-1.5302
K58114536	rasagiline	Monoamine oxidase inhibitor	-1.5257
K87737963	CYT-387	JAK inhibitor	-1.5207

## Discussion

Recurrent pregnancy loss (RPL) is a distressing disorder that results in significant physical and psychological harm to approximately 5% of women in the reproductive age group (32). Despite the continuous efforts of researchers in working on RPL (33, 34), the etiology remains unexplained in nearly half of the cases, and only empirical treatment options are available (3). Sporadic miscarriage has been described as a distinct disease, primarily characterized by the inability of abnormal embryos to progress to a viable state (35).

In recent years, new ideas have suggested that the decidual acts as a biosensor for embryonic quality, and the disruption of decidual may predispose to miscarriage. These novel insights into the underlying mechanisms of miscarriage provide new opportunities for effective interventions in cases of recurrent pregnancy loss (36).

Currently, the absence of reliable biomarkers hinders early diagnosis of RPL, which further results in poor clinical outcomes. Consequently, exploring diagnostic biomarkers for RPL is crucial for effective prevention and treatment.

In this study, we performed differential expression analysis on GSE165004 and obtained 352 differential genes. The KEGG analysis revealed the predominant involvement of DEGs in Fc gamma R-mediated phagocytosis, the Fc epsilon RI signaling pathway, and several metabolic pathways. Fc gamma receptors (FcγR) belong to a class of cell surface receptors that bind to the Fc terminus of antibodies, generating signals in cells primarily through their ITAM activation sequence (37). These receptors participate in various immune system actions, including phagocytosis, release of inflammatory mediators, and cytotoxicity in antibody-dependent cells (38, 39). Fc epsilon RI can regulate mast cell and basophil activation and is involved in IgE-mediated antigen presentation (40, 41).

Furthermore, KEGG and GSEA results indicated metabolic pathways, including Purine metabolism and Glycerophospholipid metabolism, demonstrated a significant correlation with RPL. It can be speculated that immune regulation and metabolic pathway abnormalities may exist in RPL, consistent with previous studies on RPL (32, 42).

Subsequently, WGCNA analysis was conducted, and the turquoise module, which contained 104 DEGs, showed the strongest association with RPL. Three biomarkers of RPL (WBP11, ACTR2, NCSTN) were screened using LASSO, SVM-RFE, and random forest. The findings were validated using RT-qPCR in clinical tissue samples. The experimental results were consistent with the findings of data analysis, in which the expression of WBP11 and ACTR2 were down-regulated, while the expression of NCSTN showed up-regulation in RPL tissues.

The predictive capability of hub genes in relation to RPL was assessed using ROC curves. The AUCs of the ROC curves, based on ANN model constructed with the three hub genes, were 1.00 and 0.74 in the test set GSE165004 and validation set GSE183555, indicating their strong diagnostic efficacy. We speculated that the limited sample size in the validation set might impact the accuracy of the ANN model. This highlights the necessity to increase the sample size for future studies.

WW structural domain binding protein 11(WBP11), is a nuclear protein that has been linked to various congenital developmental abnormalities and plays a crucial role in embryonic development (43). Additionally, Lina Wang et al. (44) demonstrated the significant impact of WBP11 on the proliferation and migration of gastric cancer cells. In our study, the expression of WBP11 was downregulated in RPL and could be used as a diagnostic marker for RPL.

ACTR2, also known as ARP2, encodes a protein that is a major component of the ARP2/3 complex, which promotes actin polymerization in the nucleus and thus regulates gene transcription and DNA damage repair (45). ACTR2 is highly expressed in hepatocellular carcinoma and diffuse large B-cell lymphoma and is associated with poor prognosis (46, 47). Additionally, ACTR2 has been reported as a diagnostic marker for primary thrombocythemia.

NCSTN is a protein-coding gene that encodes a transmembrane glycoprotein within the multiprotein γ-secretase complex. Mutations in this gene are ass ociated with familial hidradenitis suppurativa (48). NCSTN can affect the Notch1 and AKT signaling pathways and is involved in the development of hepatocellular carcinoma (49).

In the study by Peiru Wei et al. (10), macrophage-related genes were screened using single-cell sequencing, followed by bioinformatics analysis to identify diagnostic genes for RM. In contrast, in our study, genes in the most important modules related to RPL were screened by analyzing GSE165004 with WGCNA, and hub biomarkers were identified using multiple machine-learning algorithms. Furthermore, in the immune cell infiltration analysis, we observed a difference in the proportion of macrophages between RPL and controls as well. Our study and that of Peiru Wei are distinct yet share similarities, both studies’ results indicate that ACTR2 and NCSTN can serve as diagnostic markers for RPL. This finding further confirms the potential diagnostic value of these two genes in RPL. Possibly due to different screening criteria and analytical methods, both of us screened and validated other biomarkers as well. In conclusion, the value of these hub genes in RPL requires more experimental exploration.

Several studies have indicated that RPL may result from the disruption of maternal-fetal immune tolerance (50). Paternal antigens and even antigens from the gamete donor can be expressed by the fetus, and the maternal immune system’s response to fetal antigens may be implicated in the pathogenesis of certain RPL cases (51). Abnormalities in NK cells, T cells, B cells, and macrophages have been observed in the uterine endometrium of patients with recurrent miscarriage (52, 53).

Macrophages constitute the second largest population of immune cells, yet their role during pregnancy remains poorly understood. M1 and M2 macrophages are involved in vascular formation and immune suppression at the maternal-fetal interface (54). Macrophages in the decidual layer during pregnancy serve to protect the embryo from phagocytosis and infection (55). Abnormal recruitment and differentiation of macrophages are closely associated with RPL (56). Decidual macrophages play a crucial role in the process of pregnancy, and their dysfunction can lead to the occurrence of RPL (57). Zhao et al. (58) analyzed RPL dataset and macrophage polarization dataset, with a specific focus on URSA-associated macrophage markers and intercellular communication mechanisms. They further explored the correlation between macrophage polarization and URSA. The hub genes obtained in their study showed some discrepancies with our results, which could be attributed to different focus within the WGCNA. We focused on modules associated with RPL, while Zhao et al. placed more emphasis on genes related to macrophage polarization. In this study, CIBERSORT analysis was used to investigate immune cell infiltration in RPL, revealing low levels of infiltration by both M1 macrophages and M2 macrophages in RPL patients (**Figure 8A**). We further analyzed the correlation between hub genes and immune cells in RPL and found that three hub genes were associated with macrophages. This indicates a potential association between abnormal expression of hub genes and dysfunction of immune cell infiltration in RPL. In future research, it is essential to investigate the potential co-expression of hub genes between these two studies and design experiments to explore the regulatory relationship of hub genes with macrophages during recurrent miscarriage.

In the pan-cancer analysis, abnormal expression of three hub genes has been observed in multiple malignant tumors, and their expression is closely associated with overall survival in several cancers. This suggests that these hub genes play crucial roles in various biological processes, and conducting further research on them would contribute to a better understanding of disease occurrence and progression. Moreover, we successfully screened several small molecule drugs using the Cmap database, and these compounds showed a strong negative correlation with the DEGs of RPL. This finding suggests that these drugs may have significant therapeutic value for the future treatment of RPL.

In this study, we conducted various advanced bioinformatics methods and performed experiments, and finally identified three diagnostic biomarkers for RPL. This finding may be of potential value in the diagnosis and further research of RPL. However, the limited number of cases in both the GEO dataset and the experimental validation could amplify individual variations and introduce potential biases in the results. Therefore, subsequent research should focus on validating the findings in larger cohorts to enhance the generalizability of the research outcomes. Additionally, additional cell-based and animal experiments should be designed to further validate the biological functions of the hub genes.

## Conclusion

In conclusion, WBP11, ACTR2, and NCSTN were identified as diagnostic biomarkers of RPL, and these three hub genes are closely associated with various malignancies. Meanwhile, there is an imbalance in immune cell infiltration in RPL. Our study provides new insights into the mechanisms underlying RPL and the potential development of therapeutic targets for this condition.

## Data availability statement

The codes presented in the study are publicly available. This data can be found here: https://github.com/Weihouye/bioinformatics-analysis.git.

## Ethics statement

The studies involving humans were approved by the Research Ethics Committee of the First Affiliated Hospital of Guangxi Medical University. The studies were conducted in accordance with the local legislation and institutional requirements. The participants provided their written informed consent to participate in this study.

## Author contributions

LP, CW, and JC designed the study. CW and YW downloaded and analyzed the data. XT and JC wrote the manuscript and prepared the figures and tables. SL, XT, and ZZ edited the manuscript. All authors contributed to the article and approved the submitted version.
