# Supplementary figures and images for: Identification and verification of diagnostic biomarkers in recurrent pregnancy loss via machine learning algorithm and WGCNA
Source: Front Immunol. 2023 Aug 25;14:1241816. doi: 10.3389/fimmu.2023.1241816 (PMC10485775; doi:10.3389/fimmu.2023.1241816)

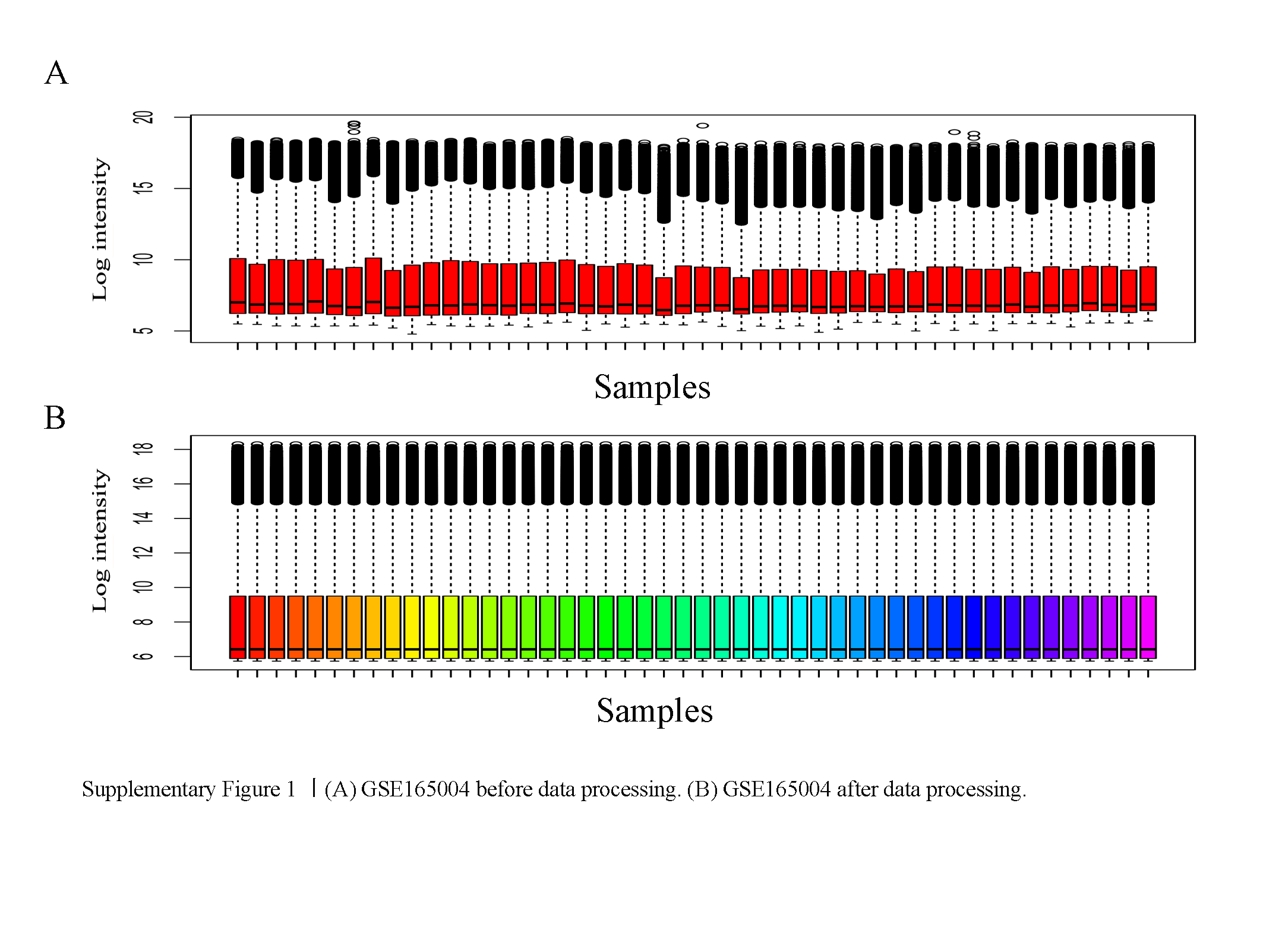

Supplement: Supplementary file 1 [file Image_1.tif]

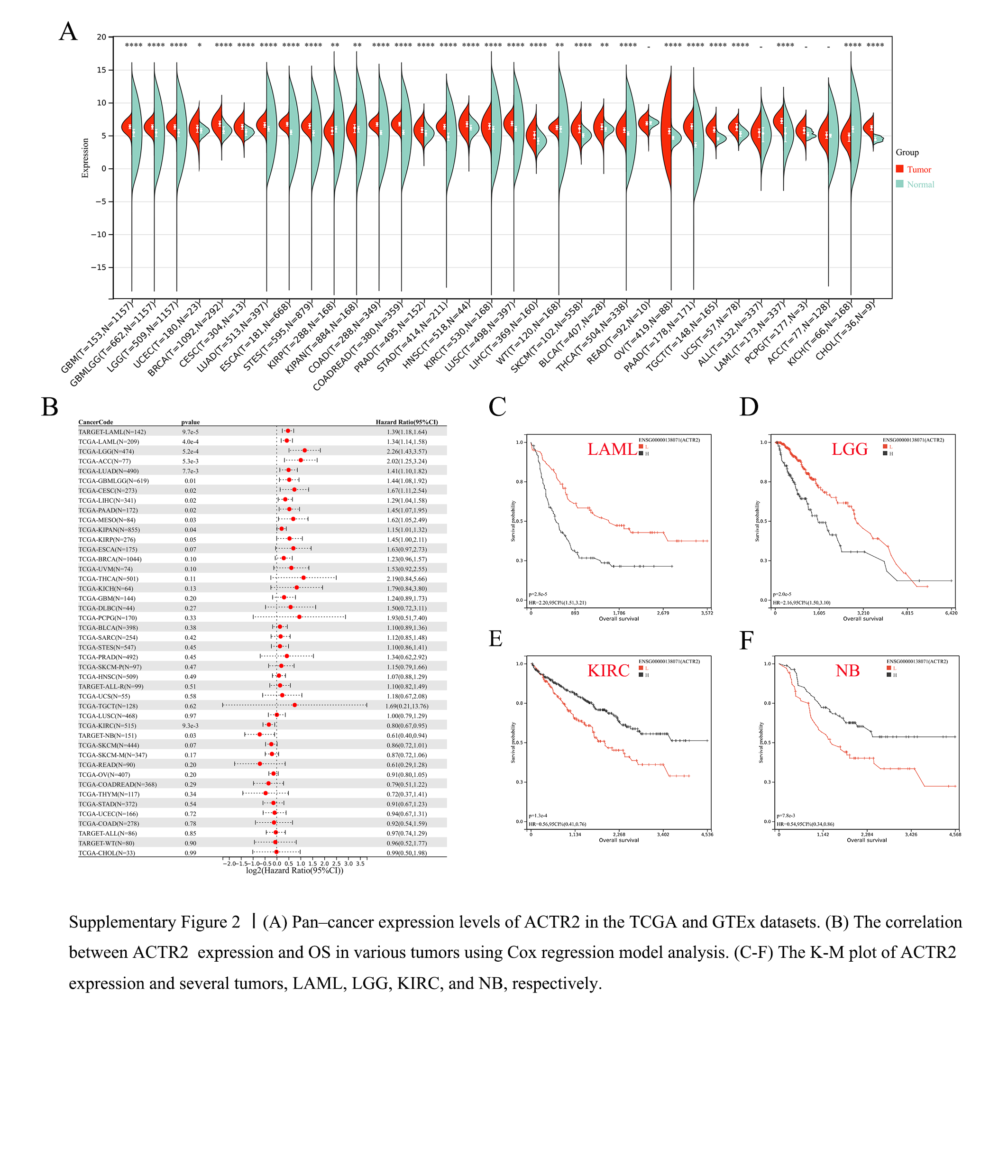

Supplement: Supplementary file 2 [file Image_2.tif]

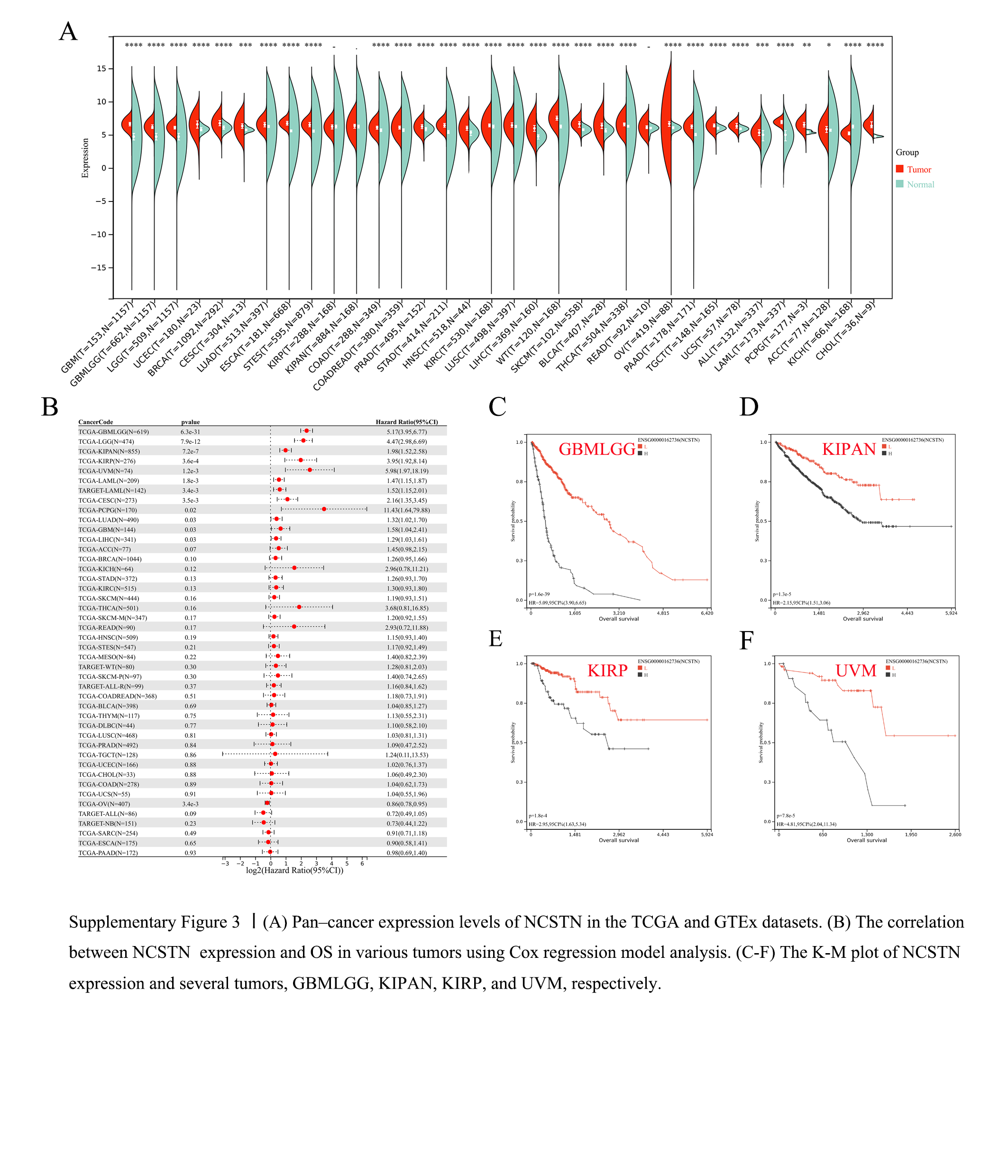

Supplement: Supplementary file 3 [file Image_3.tif]
